# High-throughput drug library screening identifies colchicine as a thyroid cancer inhibitor

**DOI:** 10.18632/oncotarget.7890

**Published:** 2016-03-03

**Authors:** Le Zhang, Zhaoying Yang, Letizia Granieri, Adrian Pasculescu, Alessandro Datti, Sylvia L. Asa, Zheli Xu, Shereen Ezzat

**Affiliations:** ^1^ Department of Breast Surgery, China-Japan Union Hospital, Jilin University, Changchun, Jilin, P.R. China; ^2^ Ontario Cancer Institute and The Endocrine Oncology Site Group, Princess Margaret Hospital, University Health Network, Toronto, ON, Canada; ^3^ SMART Laboratory for High-Throughput Screening Programs, Lunenfeld-Tanenbaum Research Institute at Mount Sinai Hospital, Toronto, ON, Canada; ^4^ Department of Agricultural, Food, and Environmental Sciences, University of Perugia, Perugia, Italy; ^5^ Departments of Laboratory Medicine and Pathobiology, Toronto, ON, Canada; ^6^ Departments of Medicine, University of Toronto, Toronto, ON, Canada

**Keywords:** thyroid cancer, colchicine, BRAF, BRAF resistance, high throughput drug screening

## Abstract

We employed a high-throughput drug library screening platform to identify novel agents affecting thyroid cancer cells. We used human thyroid cancer cell lines to screen a collection of approximately 5200 small molecules with biological and/or pharmacologial properties. Parallel primary screens yielded a number of hits differentially active between thyroid and melanoma cells. Amongst compounds specifically targeting thyroid cancer cells, colchicine emerged as an effective candidate. Colchicine inhibited cell growth which correlated with G2 cell cycle arrest and apoptosis. These effects were hampered through inhibition of MEK1/2 and JNK. In contrast, inhibition of p38-MAPK had little effect, and AKT had no impact on colchicine action. Systemic colchicine inhibited thyroid cancer progression in xenografted mice. These findings demonstrate that our screening platform is an effective vehicle for drug reposition and show that colchicine warrants further attention in well-defined clinical niches such as thyroid cancer.

## INTRODUCTION

Inhibition of the BRAF^V600E^ oncoprotein by small-molecule drugs such as PLX4032 (vemurafenib) is highly effective in the treatment of melanoma [1]. However, thyroid cancer patients who are radioiodine refractory and harbor the same BRAF^V600E^ oncogenic mutation, show variable responses to this class of inhibitors [2, 3], suggesting a lack of drug selectivity. Alternatively, underlying drug resistance might conceivably result from additional MAPK pathway alterations [4]. Accordingly, epidermal growth factor receptor (EGF-R) inhibitors appear to provide synergy with BRAF inhibitors in multiple BRAF-mutant cancers [5]. Although such drug combinations may show a more effective action, the potential for increased toxicity and economic implications can limit clinical feasibility. Additionally, recent evidence identified a role for the AKT pathway in mediating resistance to BRAF inhibition [6].

Prompted by these limitations, we set out to identify existing compounds active in thyroid cancer cells and explore their mechanism of cytotoxicity. Recognized for their BRAF^V600E^ mutation and sensitivity to BRAF inhibition [7], melanoma cells were included in our drug screens as a reference. Fully-automated screens by integrated robotics were run in parallel to employ a collection of nearly 5200 small molecules that were assembled to include most FDA-approved drugs and a large array of compounds with known biological and biochemical properties. This screening platform, purposely aimed at facilitating drug reposition strategies and mechanistic studies towards target validation clearly highlighted the potential of colchicine in thyroid cancer.

## RESULTS

### High-throughput screening and hit selection

**Figure 1:**
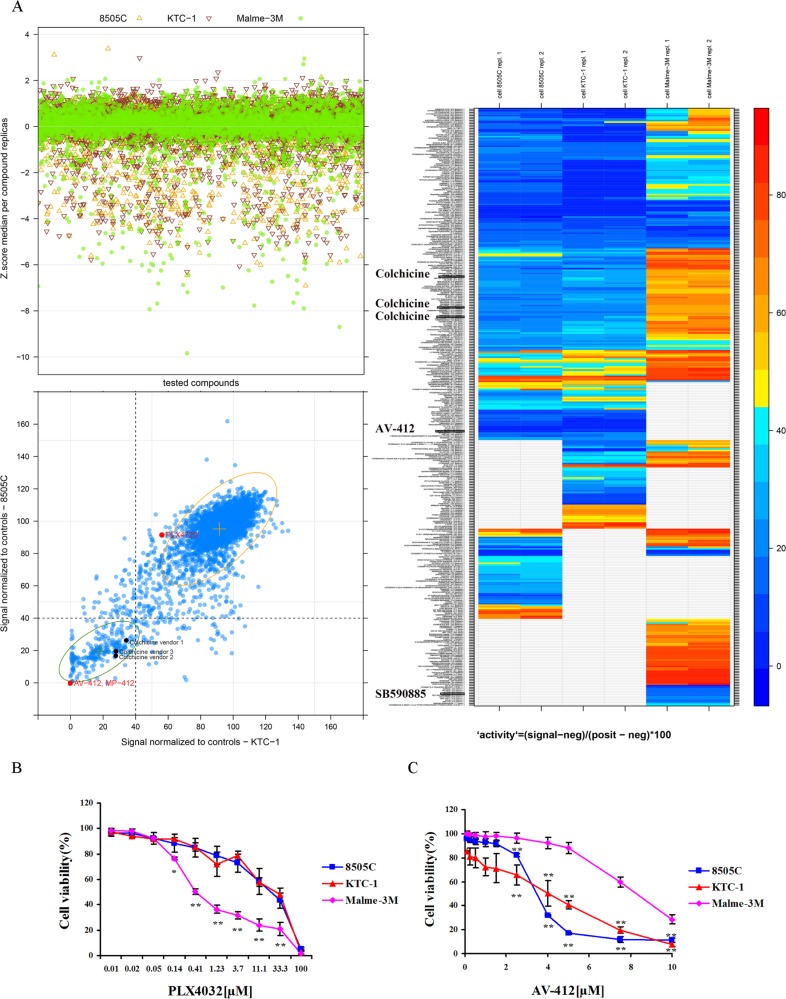
High-throughput drug library screening BRAF-mutant thyroid cancer 8505C and KTC-1 cells and melanoma cells (Malme-3M) were used as biological sources to screen, under high-throughput conditions, a multi-vendor collection of ∼5200 chemical entities with known pharmacological and biological properties. (**A**) Top left panel shows Z-score values obtained from parallel screens of cells. Bottom left panel displays a comparative chart filled with control-based normalized data resulting from the screen of thyroid cells. Samples with less than or equal to 40% viability are highlighted. Control-based normalization is calculated as follows: 100 (signal-negative control)/(positive control-negative control). PLX4720 is a BRAF inhibitor. A heat map of drug effects on cell viability is shown on the right. Red indicates highest and blue reflects lowest cell viability. AV-412 is an EGF-R inhibitor, and SB590885 is a BRAF inhibitor. (**B**) The dose-dependent effect of the BRAF inhibitor PLX4032 and (**C**) The EGF-R inhibitor AV-412 on cell viability after 48 hrs incubation. Results are expressed as a percentage in relation to positive control (100%) treated with vehicle alone, and reported as the means ± SD of three independent experiments performed with four replicates. *p < 0.05; **p < 0.01 comparing melanoma with thyroid cancer cells at each indicated dose.

To identify small molecules that can inhibit the viability of thyroid cancer cells, we developed a screening platform using 8505C and KTC-1 cells that harbor the BRAF^V600E^ mutation and, as a reference for a non-thyroidal BRAF mutant phenotype, human melanoma Malme-3M cells. The screening libraries included approximately 5200 small molecules selected to facilitate drug reposition and mechanistic studies. Primary screening data were normalized by Z-score transformation as shown in Figure 1A. The screening procedure identified a BRAF inhibitor active in melanoma Malme-3M cells (Figure 1B) (IC50 = 0.85 ± 0.19 μM) but virtually ineffective in thyroid 8505C and KTC-1 cancer cells (IC50 > 10 μM, not shown). In contrast, the EGF-R inhibitor AV-412 showed inhibitory effects on thyroid 8505C and KTC-1 cells with reduced effects on melanoma Malme-3M cells (Figure 1C). These findings provided evidence of the platform's ability to correctly identify drugs of recognized activity in a cell-contextual manner.

### Colchicine inhibits thyroid cancer cells

**Figure 2:**
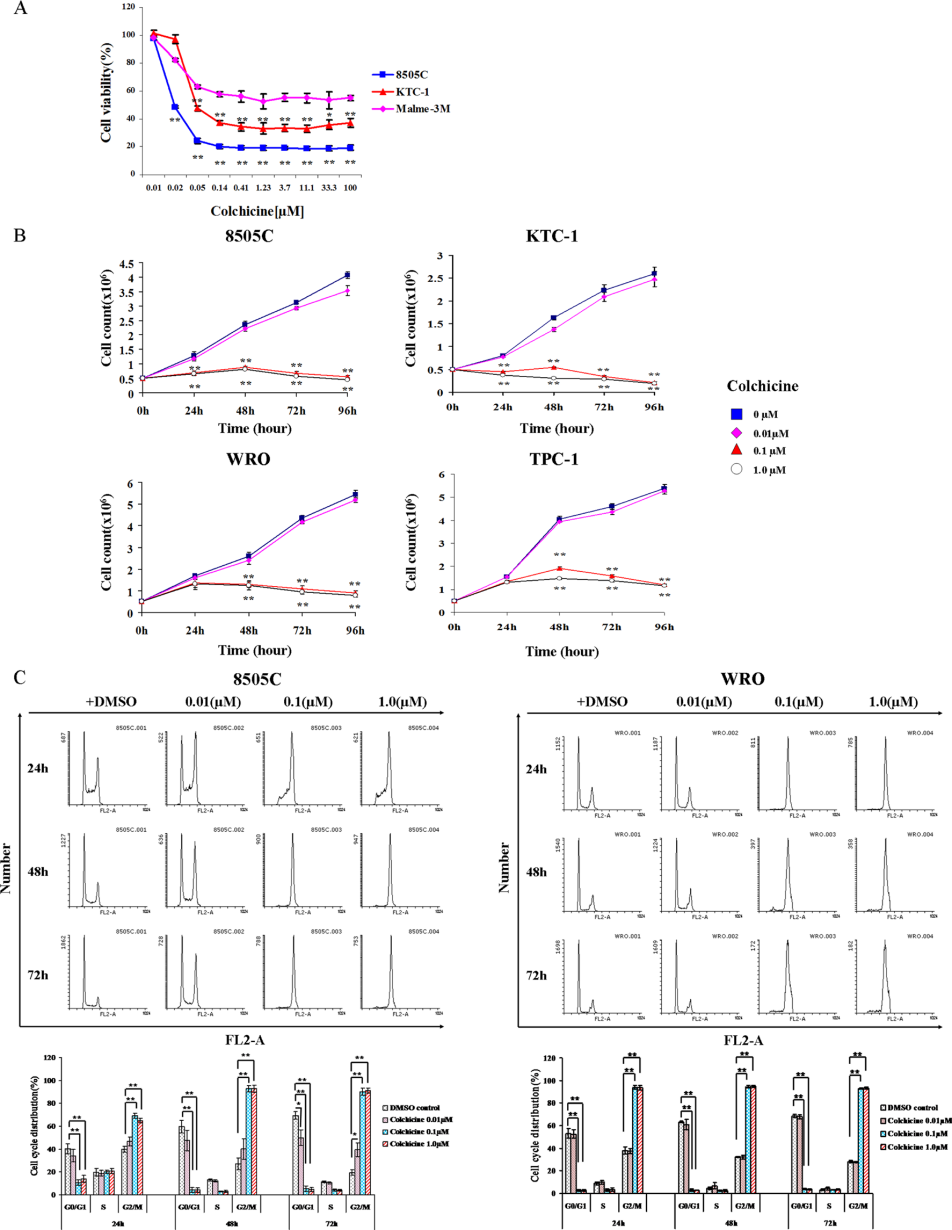
Validation of colchicine as an inhibitor of thyroid cancer cells (**A**) BRAF-mutant thyroid (8505C and KTC-1) and melanoma (Malme-3M) cells were treated in the presence of increasing doses of colchicine for 48 hrs and assessed for cell viability. *p < 0.05; **p < 0.01 comparing melanoma with thyroid cancer cells at each indicated dose. (**B**) Cell density was also monitored in two additional thyroid cancer cell lines that are BRAF-WT (WRO and TPC-1). Values are means ± SD of three independent experiments. **p < 0.01 comparing colchicine with DMSO control at the same time point. (**C**) Cell cycle analysis was monitored by flow cytometry using propidium iodide (PI) dye staining. After 24 hrs of serum starvation, cells were treated with vehicle (DMSO) or colchicine at different doses and times as indicated. Cell cycle profile is estimated by gating histograms generated with the FL2-area variable. The percentage of cells is shown as the mean ± SD of three independent experiments immediately below. *p < 0.05; **p < 0.01 comparing indicated dose of colchicine with DMSO control at the same time point and cell cycle phase.

From the initial screens, we selected colchicine, which was present in 3 different locations within the screening library, as one of the top hits (Figure 1A). Validation testing demonstrated the ability of colchicine to decrease 8505C and KTC-1 thyroid cancer cell viability with IC50 of 0.02 ± 0.00 μM and 0.44 ± 0.17 μM, respectively, whereas a much lower activity was displayed in melanoma cells (Figure 2A). Importantly, these findings were also extended to other thyroid cancer cell lines including WRO and TPC-1 cells (Figure 2B).

### Colchicine induces growth arrest of thyroid cancer cells at G2/M phase

To examine the mechanisms underlying growth inhibition of colchicine, we monitored cell cycle phase progression by flow cytometry. Figure 2C demonstrates the impact of colchicine on increasing the proportion of 8505C and WRO cells in G2/M phase, and shows a markedly diminished entry of cells into the G1 phase.

### Colchicine induces apoptosis of thyroid cancer cells

**Figure 3:**
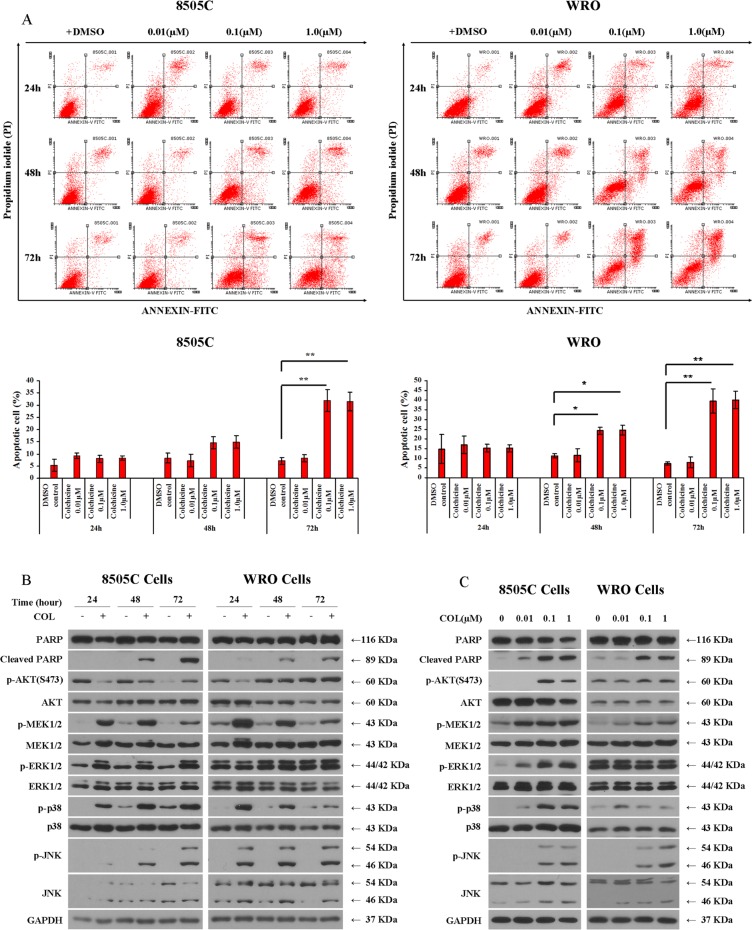
Impact of colchicine on thyroid cancer cell apoptosis (**A**) After 24 hrs serum starvation, 8505C and WRO cells were incubated with vehicle (DMSO) or colchicine as shown. The apoptotic cell population was detected by Annexin V-FITC and PI staining using flow cytometry. The percentage of apoptotic cells is shown as the mean ± SD of three independent experiments immediately below. (**B**) 8505C and WRO cells were treated with or without 0.1 μM of colchicine and incubated for different times as shown prior to Western blotting. (**C**) 8505C and WRO cells were treated for 72 hrs with the indicated doses of colchicine prior to Western blotting. *p < 0.05; **p < 0.01 comparing indicated dose of colchicine with DMSO control at the same time point.

We next assessed the mode of colchicine-mediated thyroid cell death. Externalization of phosphatidylserine, an early marker of apoptosis detected by Annexin V, and late marker of apoptosis detected by PI, were observed by flow cytometry in live cells treated with variable concentrations of colchicine (0.01–1.0 μM) across different time points (24–72 hrs) (Figure 3A). Further, we observed that the effect of colchicine correlated with PARP cleavage in a time- (Figure 3B) and dose-dependent manner (Figure 3C), as detected by Western blotting. Importantly, the pro-apoptotic action of colchicine was accompanied by the activation of multiple signaling pathways. In 8505C cells, we noted increased phosphorylation of the MAP kinases MEK/ERK, p38, and JNK. Further, despite an early inhibitory impact detected after 24 hrs treatment, AKT phosphorylation was increased by 72 hrs in both cell types (Figure 3B). In WRO cells, we also noted increased MEK, p38, and JNK phosphorylation in response to colchicine treatment, while elevated pERK levels remained unaffected (Figure 3B, 3C).

**Figure 4:**
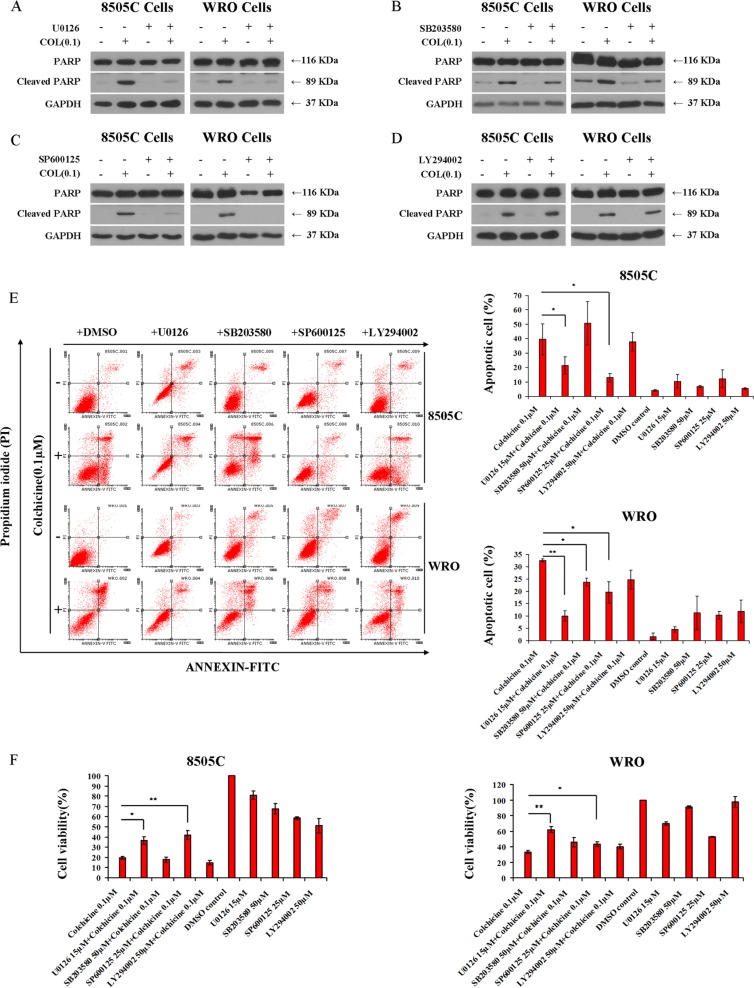
Involvement of MAPK in colchicine-induced thyroid cancer cell apoptosis Western blotting of 8505C and WRO cells to assess PARP cleavage under different treatment conditions. After 24 hrs serum starvation, cells were incubated with vehicle (DMSO), 15 μM of the MEK/ERK1/2 inhibitor U0126 (**A**), 50 μM of the p38 inhibitor SB203580 (**B**), 25 μM of the JNK inhibitor SP600125 (**C**), and 50 μM of the PI3K inhibitor LY294002 (**D**) in the presence or absence of 0.1 μM of colchicine for 72 hrs. (**E**) Cells were treated as shown in panels A–D and subjected to flow cytometry analysis for evaluation of Annexin V-FITC and PI staining. Results were interpreted as follows: (Annexin V^−^, PI^−^ : viable cells); (Annexin V^+^, PI^−^ : cells undergoing early apoptosis); (Annexin V^+^, PI^+^: cells in late apoptosis); and (Annexin V^−^, PI^+^: necrotic cells). The percentage of apoptotic cells is shown as the mean ± SD of three independent experiments. (**F**) Effects of MAPK and PI3K inhibitors on cell viability under the treatment conditions shown. Cell viability was monitored by the Alamar Blue assay. Data are shown as the mean ± SD of three experiments. *p < 0.05; **p < 0.01 comparing colchicine with combination treatments of indicated inhibitors.

To study the significance of these pathway alterations in relation to colchicine mode-of-action, we co-incubated colchicine with a variety of chemicals known to impact specific cell signaling routes. Inhibition of MEK1/2 with U0126 virtually abolished the apoptotic effect of colchicine, as shown by absence of PARP degradation (Figure 4A), reduced apoptotic cell fraction (Figure 4E), and rescue of cell viability (Figure 4F). Similarly, we noted the ability of the JNK inhibitor SP600125 to diminish colchicine-induced apoptosis (Figure 4C, 4E) and rescue cell viability (Figure 4F). In contrast, the effect of the p38 inhibitor SB203580 was less evident on PARP degradation in WRO cells (Figure 4B), and it only partially rescued these cells from apoptosis (Figure 4E) with minimal impact on cell viability (Figure 4F) in response to colchicine treatment. Additionally, SB203580 failed to rescue colchicine-induced PARP degradation (Figure 4B), apoptosis (Figure 4E), or viability (Figure 4F) of 8505C cells. Moreover, the AKT inhibitor LY294002 had no measureable impact on colchicine action in either cell type including apoptosis (Figure 4D, 4E) and cell viability (Figure 4F), thus ruling out AKT involvement in colchicine action in these cells.

**Figure 5:**
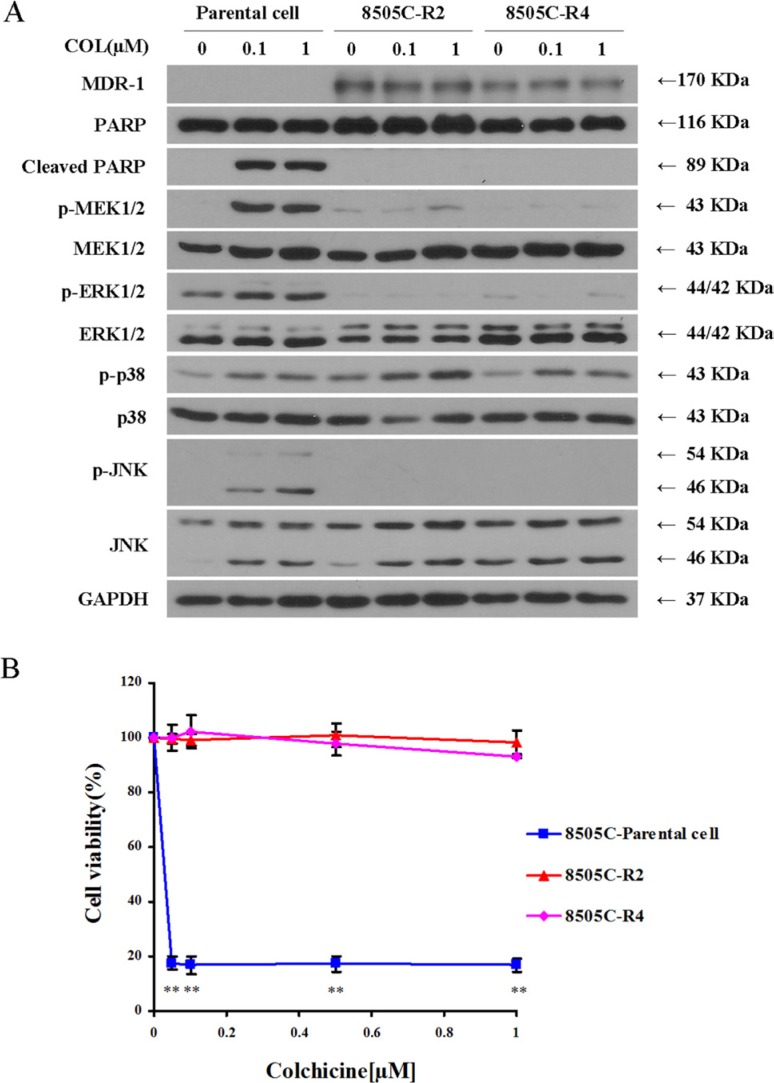
Colchicine–resistant thyroid cancer cells (8505C) show reduced MEK/ERK1/2 and JNK activity (**A**) Parental 8505C cells and two resistant clones 8505C–R2 and 8505C–R4 were subjected to serum starvation for 24 hrs. Cells were then treated with vehicle (DMSO) or colchicine (0.1 μM and 1 μM) for 72 hrs, and subjected to Western blotting for assessment of MDR-1 expression, MAPK activation, and PARP cleavage. (**B**) Cell viability by Alamar Blue staining of parental 8505C cells and two resistant clones 8505C–R2 and 8505C–R4 was performed in the presence of increasing concentrations of colchicine incubated for 72 hrs as indicated. The percentage of viable cells is shown as the mean ± SD of three independent experiments. **p < 0.01 comparing parental cells with resistant clones at each indicated dose.

To further distinguish between the roles of different MAP kinases on colchicine action, we generated colchicine-resistant 8505C cells. Confirmation of drug resistance was demonstrated by expression of the MDR-1 marker (Figure 5A), the absence of PARP degradation (Figure 5A), and the lack of any impact on cell viability following colchicine treatment (Figure 5B). Compared with their sensitive parental cells, colchicine-resistant 8505C cells continued to exhibit measureable p38 phosphorylation responses, but failed to stimulate MEK, ERK1/2, or JNK (Figure 5A).

### Colchicine inhibits thyroid tumor growth *in vivo*

**Figure 6:**
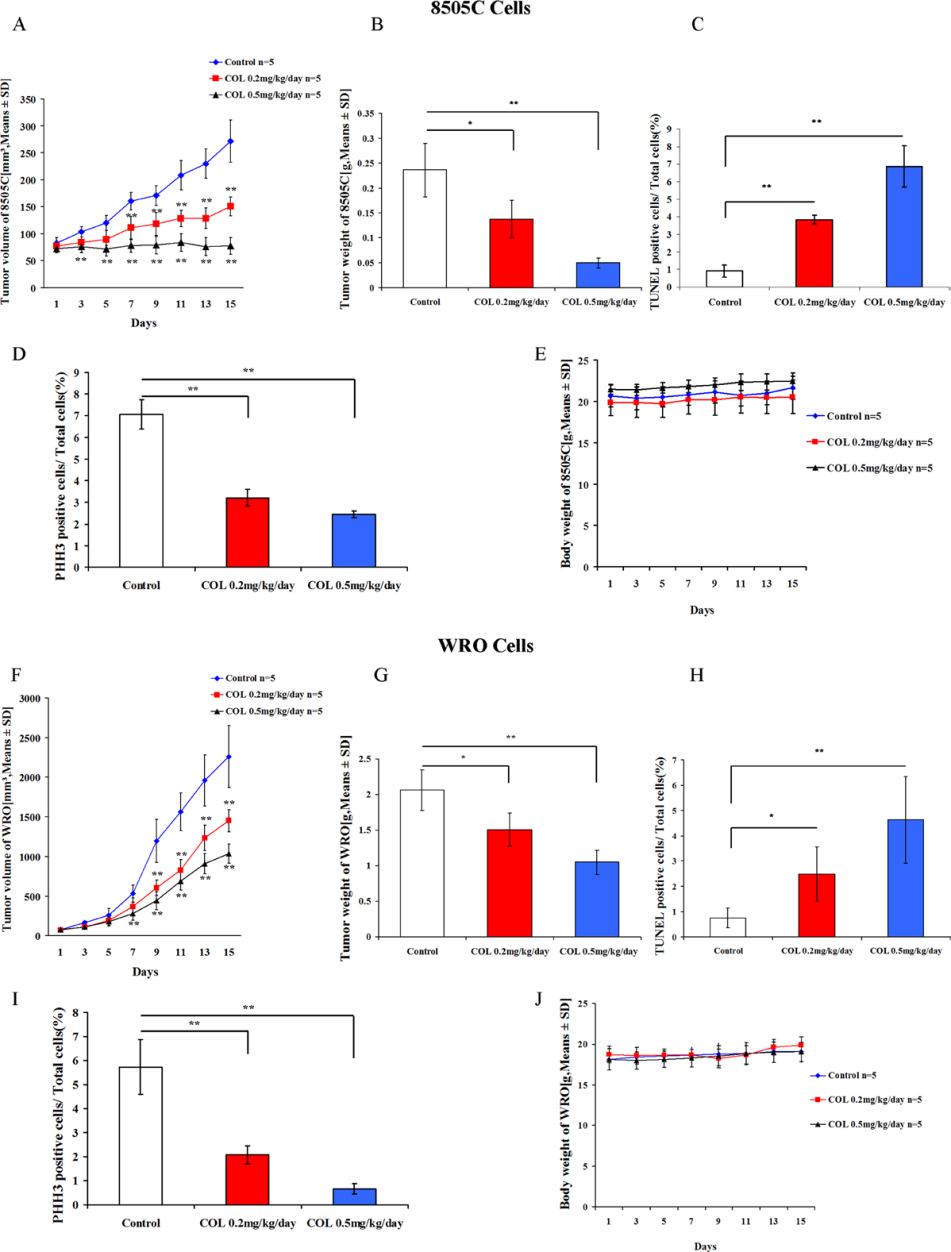
Colchicine activity in thyroid cancer mouse xenografts Five × 10^6^ thyroid cancer 8505C and WRO cells were introduced subcutaneously into female SCID mice. Once a tumor was palpable, animals were treated for two weeks with the indicated doses of colchicine or DMSO vehicle control. Results shown include the following evaluations: Tumor volumes (**A**, **F**), tumor weight (**B**, **G**), body weight (**E**, **J**), TUNEL staining of xenografted tissue to assess apoptosis (**C**, **H**), and PHH3 staining to visualize mitotic phase (**D**, **I**). Values represent the means ± SD with 5 animals in each experimental group. *p < 0.05; **p < 0.01 comparing colchicine with DMSO control at each indicated dose.

To determine whether the effects of colchicine observed *in vitro* had an impact on tumor growth *in vivo*, we tested this drug in thyroid 8505C and WRO cancer cell mouse xenografts. These studies revealed the ability of colchicine to significantly reduce 8505C tumor volume and tumor weight (Figure 6A, 6B). Comparable results were also recapitulated in the WRO cell xenografts (Figure 6F, 6G). Further, TUNEL staining identified a significant impact on apoptosis in both animal models (Figure 6C, 6H). PHH3 staining also demonstrated the ability of colchicine to reduce the proportion of proliferating malignant cells in both cell line xenografts (Figure 6D, 6I). Finally, we observed that colchicine administration was well-tolerated in mice with no adverse impact on body weight (Figure 6E, 6J), and overall health (not shown).

## DISCUSSION

The relevance to thyroid cancer of the MAPK pathway and of the BRAF^V600E^ mutation is now well-established [8–10]. Its diagnostic relevance has been accepted [11], however, its prognostic significance remains controversial [12, 13]. Selective pharmacologic targeting of this kinase in thyroid cancer remains limited but encouraging [7, 14]. From a clinical perspective, the appreciation of discrepant responses amongst BRAF mutant cancers [15, 16] has highlighted the need for additional therapeutic strategies.

In this report, colchicine was selected using a high-throughput screening campaign, and our findings, altogether, point to its therapeutic impact in thyroid cancer. Notably, high-throughput (HT) drug screens were rarely employed for thyroid cancer. In this regard, a HEK 293-based HT assay was previously shown to identify cyclic AMP agonists of the TSH receptor [17] and, more recently, a different platform yielded EGFR/HER2 and HDAC inhibitors as possible therapeutics [18].

Colchicine represents a vinca alkaloid clearly recognized for its ability to bind tubulin [19]. Microtubules have long been considered as an ideal target for anti-cancer drugs because of their essential functions in mitosis and, in particular, the formation of the dynamic spindle apparatus. In this regard, colchicine inhibits tubulin polymerization by blocking the cell cycle at the G2/M phase and triggering apoptosis [20]. While such an impact may lead to toxic effects, orally-administered colchicine is a well-established, safe treatment [21]. Thus, it may be reasonable to consider repositioning of colchicine as a lead compound, alone or in combination with other drugs.

Here, we show that colchicine exhibits cytotoxicity against thyroid cancer cell lines in the nanomolar concentrations. Our findings implicate multiple MAP kinases in colchicine-induced apoptosis. Consistent with our current findings, earlier studies suggested the involvement of JNK as a putative MAPK target in thyroid cancer cell growth [22]. In contrast, the ability of colchicine-resistant cells to support p38 phosphorylation raises questions about requirements of this MAPK in colchicine-induced thyroid cancer cell apoptosis. Our results are partially supported by earlier findings where colchicine-induced neuronal toxicity was linked to JNK and p38-MAPK, but not with ERK1/2-MAPK activation [23]. We propose that recruitment of multiple MAPKs affords colchicine, as shown here, broader activity across BRAF-mutant and BRAF-WT thyroid cancer cells.

Recently, allocolchicines and other analogues have shown potentially promising effects in cancer cells. Of note, the (S)-3, 8, 9, 10-tetramethoxyallocolchicine derivative, while showing effective chemotherapeutic properties against pancreatic cancer cells, did not perturb tubulin polymerization, suggesting involvement of alternative targets [24]. It is therefore possible that colchicine analogues display reduced toxicity profiles, thus boosting their suitability for the treatment of thyroid cancer.

## MATERIALS AND METHODS

### Chemicals and reagents

The EGF-R inhibitor AV-412 (MP-412) was obtained from AdooQ Bioscience, colchicine was obtained from Sigma-Aldrich. The BRAF inhibitor PLX4032, the MEK1/2 inhibitor U0126, the p38 inhibitor SB203580, the JNK inhibitor SP600125, and the PI3K inhibitor LY294002 were obtained from Selleck Chemicals (Houston, TX). All drugs were dissolved in dimethyl sulfoxide (DMSO) and stored at −20°C.

### Cell lines and culture

The human thyroid carcinoma–derived cell lines 8505C, KTC-1 (Cell Resource Center for Biomedical Research, Sendai, Japan) and WRO (G. Juilliard, UCLA, Los Angeles) were maintained in RPMI-1640 (Gibco) supplemented with 10% FBS, 2 mmol/L L-glutamine, 1 mmol/L sodium pyruvate, 1 × non-essential amino acids, streptomycin sulfate (100 U/ml), and penicillin (100 μg/ml). TPC-1 (S. Jiang, Ohio State University) thyroid cancer cells were cultured in Dulbecco's modified Eagle's medium (DMEM) supplemented with 5% FBS, and 2 mmol/L L-glutamine. The human melanoma carcinoma–derived cell line Malme-3M was cultured in DMEM with 10% FBS and 1 mmol/L sodium pyruvate. All cell lines were grown and maintained at 37°C, 95% humidity, and 5% CO_2_. Cell lines were assembled in 2006 and authenticated by short tandem repeats. Cell counts were performed with a Beckman Coulter counter (Fullerton) while cell viability was determined by Alamar Blue (Invitrogen) staining. Development of colchicine-resistant sub-lines was performed by continuous exposure to colchicine using an initial concentration of 0.01 μmol/L that was increased in a step-wise fashion to 1.0 μmol/L. Resistant cell lines were maintained in media containing 1.0 μmol/L colchicine for more than 6 months.

### High-throughput screening (HTS)

High-throughput screens were performed in the SMART Laboratory for High-Throughput Screening Programs of the Lunenfeld-Tanenbaum Research Institute (Mount Sinai Hospital, Toronto). Assays were fully automated on a Dimension 4, modular platform (Thermo Fisher Scientific) equipped with a Biomek FX liquid handler. Screening compounds (*n*∼5200) were acquired from several vendors re-assembled to form a unique collection suitable for drug reposition and mechanistic studies aimed at target identification and validation. Compounds included FDA-approved drugs, drug candidates with history in human clinical trials, and chemical entities (including 320 protein kinase inhibitors) with known impact on key molecular players, signaling pathways, and more general biological processes. Chemicals were obtained from Prestwick Chemical (Prestwick Chemical Library), Tocris Bioscience, MicroSource Discovery Systems (Spectrum Collection), BioFocus (NIH Clinical Collection), and the Ontario Institute for Cancer Research (protein kinase inhibitors). Cells were seeded in 384-well plates in a total volume of 40 μL/well. On the following day, screening compounds (1mmol/L in DMSO) were robotically pinned (200 nL) into assay wells to achieve a final concentration of 5 μmol/L. Equal amounts of DMSO (0.5%) were used as vehicle controls. After 48 hrs incubation, Alamar Blue was added at 10% of the volume (4 μL/well), and cell viability was read 4 hrs later using a PHERAstar plate reader (BMG Labtech).

### Flow cytometry

Approximately 2 × 10^6^ cells were suspended in 0.5 ml of phosphate-buffered saline and vortexed with 3 ml of 80% ethanol. Cells were fixed at 4°C for 1 hr, washed with phosphate-buffered saline, re-suspended in 3 ml of staining Buffer (0.2% Triton X-100, 1 mM EDTA in D-PBS), vortexed vigorously, and left at room temperature for 5 min. Cells were re-suspended in 1 ml of staining buffer containing 50 μg/ml of DNase-free RNase A and 50 μg/ml of propidium iodide (PI). Cells were stained for at least 1 hr at room temperature in the dark. Stained cells were analyzed by fluorescence-activated cell sorting (FACS) on a FACS CALIBUR4 flow cytometer.

### Cell apoptosis analysis

Annexin V-FITC staining of phosphatidylserine residues is indicative of early apoptotic events, whereas propidium iodide staining is a typical marker of late apoptosis processes. We used the Annexin V-FITC apoptosis detection kit with PI from Biolegend (San Diego, CA). Briefly, cells were washed twice with cold PBS and re-suspended in binding buffer and incubated with 5 μl of Annexin V-FITC and 5 μl of PI for 15 min at room temperature in the dark. Stained cells were then analyzed by FACS CALIBUR4 flow cytometer within 1 hr. Data were analyzed using the Flowing software 2.

### Western blotting

Cells were lysed in RIPA buffer (0.5% sodium deoxycholate, 0.1% SDS, 1% Nonidet P-40, and 1× PBS) containing proteinase inhibitors (100 μg/ml phenylmethylsulfonylfluoride, 12 μg/ml aprotinin) and 1 mM sodium orthovanadate (Sigma-Aldrich). Cell lysates were prepared and quantified for protein concentration by the Bio-Rad (Hercules, CA) method. Equal amounts of protein (50 μg) solubilized in sample buffer were separated on 10% or 12% SDS polyacrylamide gels and transferred electrophoretically onto nitrocellulose membranes. Membranes were blocked in TBS containing 0.1% Tween 20 (TBS-T) plus 5% non-fat dried milk for 1 hr at room temperature, probed with various primary antibodies (Table 1) at 4°C overnight. Membranes were washed 3 times and incubated with the appropriate secondary antibodies (anti-mouse or anti-rabbit IgG) conjugated with horse-radish peroxidase at a dilution of 1:2000 in blocking buffer for 1 hr at room temperature. Targeted proteins were visualized using an ECL chemiluminescence detection system (Amersham).

**Table 1:** List of antibodies

#	Antibody Name	Source	Manufacturer	Working Concentration
1	p-AKT (Ser473)	Rabbit	Cell Signaling Technology	WB: 1:1000
2	AKT	Rabbit	Cell Signaling Technology	WB: 1:1000
3	p-MEK1 (Ser218/222)/MEK2 (Ser222/226)	Rabbit	Upstate	WB: 1:1000
4	MEK1/2	Rabbit	Cell Signaling Technology	WB: 1:1000
5	p-ERK1/2 (Thr202/Tyr204)	Rabbit	Cell Signaling Technology	WB: 1:1000
6	ERK1/2	Rabbit	Cell Signaling Technology	WB: 1:1000
7	p-p38 (Thr180/Tyr182)	Rabbit	Cell Signaling Technology	WB: 1:1000
8	p38	Rabbit	Cell signaling Technology	WB: 1:1000
9	p-JNK (Thr183/Tyr185)	Rabbit	Cell Signaling Technology	WB: 1:1000
10	JNK	Rabbit	Cell Signaling Technology	WB: 1:1000
11	PARP	Rabbit	Cell Signaling Technology	WB: 1:1000
12	MDR-1	Mouse	Santa Cruz	WB 1:1000
13	PHH3(Ser10)	Rabbit	Upstate	IHC 1:2000
14	GAPDH	Rabbit	Cell Signaling Technology	WB: 1:1000

### *In vivo* mouse studies

Animal studies were carried out in accordance with the Canadian Council for Animal Care guidelines and approved by the Ontario Cancer Institute (OCI) Animal Care and Use Committee. Six week-old female severe-combined immunodeficient (SCID) mice were purchased from OCI (Toronto, Canada). Five × 10^6^ cells were implanted subcutaneously into the right flanks of mice. Treatment was initiated after tumor diameter had reached 5 mm followed by random allocation of animals to equalize variations in tumor volume across groups. Mice were divided into 3 groups: the control group was treated with DMSO (5%), the second group was subcutaneously treated with colchicine (0.2 mg/kg/day), and the third group was subcutaneously treated with colchicine (0.5 mg/kg/day) daily for 2 weeks. Tumor volume ([length × width^2^]/2) and bodyweight were measured every other day. Mice were sacrificed and tumors dissected and weighed. Mouse organs were fixed in 10% formaldehyde for paraffin embedding.

### Morphologic studies of mouse xenografts

To detect apoptosis, we used the terminal deoxyncleotidyl transferase TDT-mediated deoxyuridine-triphosphate nick-end labeling (TUNEL) technique (ApopTag kit, Oncor, NY). Paraffin sections were dewaxed through changes of xylene, hydrated through graded alcohols and pretreated with 1% pepsin (Sigma) in 0.01N HCl at pH 2.0 at 37°C for 15 min. Endogenous peroxidase was blocked using 3% aqueous hydrogen peroxide and endogenous biotin activity was blocked with an avidin/biotin blocking kit (Vector labs). Sections were incubated with the Biotin-nucleotide cocktail [DNA Polymerase 1 Large (Klenow) Fragment (Promega); dATP, dCTP, dGTP (Promega); Bio-11-dUTP Cedarlane 40029BT)] in at 37°C for 30 min. The reaction was visualized with the Ultra Streptavidin Horseradish Peroxidase Labeling Reagent (ID Labs Inc.) for 30 mins at room temperature and developed with freshly prepared DAB (Dako) and counterstained with Mayer's hematoxylin. To detect cell proliferation we used phosphorylated histone H3 (PHH3) staining (Upstate, NY). Sections were pretreated with citrate at pH 6.0 and the antibody was incubated at 1/2000 for one hour, non-specific immunoglobulin binding sites were blocked with normal serum. Staining was visualized with the Vectastain Universal Elite kit and DAB peroxidase substrate (Vector Laboratories). Sections were counter stained with Gill's hematoxylin. The proportion of reactive cells was scored in 6 regions from each tumor where a total of ∼1000 cells (range 550–1200) were counted.

### Statistical analyses

Data are presented as means ± SD. Statistical analyses were performed using the software package (SPSS 19.0). We used independent *t*-tests for comparison between different cell types and for different animal groups. We used paired *t*-tests for comparison of treatments at the same time point of the same cell type. *P*-values of 0.05 or less were considered statistically significant.
